# GT-SRR: A Structured Method for Social Relation Recognition with GGNN-Based Transformer

**DOI:** 10.3390/s25102992

**Published:** 2025-05-09

**Authors:** Dejiao Huang, Menglei Xia, Ruyi Chang, Xiaohan Kong, Shuai Guo

**Affiliations:** 1 College of Science, Qingdao University of Technology, Qingdao 266000, China; huangdejiao28@163.com (D.H.); changruyizuiwudi@gmail.com (R.C.); kongxiaohan616@gmail.com (X.K.); 2 College of Information and Control Engineering, Qingdao University of Technology, Qingdao 266000, China; xiamenglei0703@163.com

**Keywords:** SRR, GGNN, transformer, feature interaction modeling

## Abstract

Social relationship recognition (SRR) holds significant value in fields such as behavior analysis and intelligent social systems. However, existing methods primarily focus on modeling individual visual traits, interaction patterns, and scene-level contextual cues, often failing to capture the complex dependencies among these features and the hierarchical structure of social groups, which are crucial for effective reasoning. In order to overcome these restrictions, this essay suggests a SRR model that integrates Gated Graph Neural Network (GGNN) and Transformer. The task for SRR in this model is image-based. Specifically, the purpose of a novel and robust hybrid feature extraction module is to capture individual characteristics, relative positional information, and group-level cues, which are used to construct relation nodes and group nodes. A modified GGNN is then employed to model the logical dependencies between features. Nevertheless, GGNN alone lacks the capacity to dynamically adjust feature importance, which may result in ambiguous relationship representations. The Transformer’s multi-head self-attention (MSA) mechanism is integrated to improve feature interaction modeling, allowing the model to capture global context and higher-order dependencies effectively. By fusing pairwise features, graph-structured features, and group-level information. Experimental results on public datasets such as PISC demonstrate that the proposed approach outperforms comparison models including Dual-Glance, GRM, GRRN, Graph-BERT, and SRT in terms of accuracy and mean average precision (mAP), validating its effectiveness in multi-feature representation learning and global reasoning.

## 1. Introduction

Social relationships and interactive behaviors are fundamental to human social structures, significantly influencing societal development. SRR leverages visual data analysis to infer interpersonal relationships by examining individual attributes and interactions within images. This approach has been extensively used in fields including innovative city management [1], group emotion detection [2], behavioral analytics, and scene interpretation.

Recent advancements in deep learning and computer vision have propelled SRR research [3,4,5,6,7,8,9,10,11]. However, the complexity and diversity of social relationships, coupled with varying visual contexts and intricate semantic interrelations among features, continue to pose challenges to the accuracy and robustness of SRR systems. Existing studies have primarily focused on extracting and modeling individual, interactional, and contextual features. Techniques based on Convolutional Neural Networks (CNNs) [12] have been employed for individual feature extraction, analyzing facial expressions, attire, and postures to infer social relationships [9,13,14]. However, these methods often overlook interpersonal interactions, limiting their effectiveness in dyadic scenarios. To address this, researchers have incorporated interactional features, such as relative positioning [15,16] and proximity, recognizing their significance in reflecting social bonds. In social scenarios, individuals with intimate or familiar relationships are typically positioned in close physical proximity and tend to orient themselves toward each other, while strangers usually maintain greater interpersonal distance. However, interaction modeling approaches that rely solely on spatial or geometric features often fail to perform well in complex multi-person environments, primarily due to their limited capacity to incorporate rich contextual information.

Additionally, integrating contextual features has become prevalent in SRR research [13,14], providing environmental insights into interpersonal dynamics. For example, domestic settings often suggest intimate relationships, while professional settings imply professional associations. However, existing approaches frequently treat individual, interactional, and contextual features in isolation, lacking comprehensive modeling of their interdependencies, which hinders performance in diverse relational contexts. Moreover, research on modeling the interplay between group characteristics and dyadic relationships remains relatively underexplored. Group-level features offer insights into broader social behaviors, enriching the understanding of dyadic relationships by providing essential contextual information. For example, group activities can reveal interaction patterns and role dynamics, which are crucial for understanding relationships in complex scenarios. Ignoring group semantics leads to a fragmented understanding of dyadic relationships. Moreover, conventional SRR methods rely on single-pass reasoning, failing to capture higher-order correlations among features. GNN-based techniques [9,17,18,19] construct relational graphs to model node interactions, effectively capturing local feature relationships. However, the iterative nature of GNNs presents challenges in modeling global contexts, limiting the representation of complex semantic associations.

To address these challenges, this paper introduces a novel SRR model that effectively combines GGNN and Transformer architectures. The main contributions are as follows:

- Hybrid Feature Extraction: The model constructs relational and group nodes by leveraging multi-layer networks to extract individual features, interactional positional data, and group information. An enhanced GGNN captures the logical relationships among these nodes, while an attention mechanism dynamically weights scene features, effectively modeling complex inter-feature interactions.
- Integrated Feature Fusion Strategy: The model integrates pairwise features, relational graph data, and group characteristics. Group features supplement pairwise recognition by offering comprehensive contextual insights. A feature weighting mechanism dynamically adjusts each feature’s impact type, enhancing the model’s expressiveness and robustness, particularly in complex relational scenarios.
- Advanced Relationship Modeling with GGNN and Transformer: Building upon initial modeling, the combined GGNN and Transformer framework leverages the MSA feature of the Transformer to enhance global context modeling and conduct higher-order correlation analysis. While the GGNN effectively captures local feature relationships, the Transformer enables deep inter-feature interactions through global reasoning, ultimately improving the representation of social relationships and boosting classification performance.

The remainder of the paper is organized as follows: Section 2 reviews relevant work on Transformer-based relational modeling and SRR. Section 3 details the proposed GT-SRR framework, including hybrid feature extraction, hierarchical graph reasoning, and Transformer-based global fusion. Section 4 presents the experimental setup, implementation details, and comparative evaluations on benchmark datasets. Finally, Section 5 wraps up the paper.

## 2. Related Work

### 2.1. SRR

SRR is a pivotal research area in computer vision, focusing on inferring interpersonal interactions from visual data. This involves extracting individual features and modeling interactions, spatial configurations, and social contexts to elucidate complex social networks and interpersonal relationships.

Early approaches predominantly utilized handcrafted features, emphasizing attributes such as skin and hair color [20], as well as other appearance-related characteristics [21], primarily for identifying basic kinship relations. The advent of the People in Social Context (PISC) dataset [16] facilitated more sophisticated modeling by providing extensive data support. This led to the adoption of advanced neural structures that can extract facial, bodily, and contextual scene features to recognize intricate social relationships. For instance, Li et al. [16] employed CNNs to extract body features from segmented individual and joint regions, concurrently capturing visual context. Zhang et al. [12] introduced a multi-granularity inference framework incorporating Graph Convolutional Networks (GCNs) to model facial keypoints, significantly enhancing recognition accuracy for complex relationships. Additionally, Goel et al. [22] proposed an end-to-end neural network model capable of automatically generating social relationship graphs from input images, facilitating the inference of diverse relationship types.

The proliferation of GNNs has shifted research towards graph-based methodologies for SRR. GNNs adeptly model complex relational structures by capturing dependencies and interactions among various relationship types through graph nodes and edges. Wang et al. [23] developed a model integrating graph reasoning to enhance recognition by merging relational and environmental data. Guo et al. [24] suggested a method of social relation reasoning based on triangular constraints, introducing the Triangular Reasoning Graph Attention Network (TRGAT), which leverages attention and contrastive learning to capture high-order context and effectively improve reasoning accuracy and consistency. Li et al. [25] introduced a GNN-based social recommendation algorithm, enhancing system efficiency by learning interaction data within social graphs. Qing et al. [14] combined GGNNs and GCNs to fuse global and local information, effectively capturing dynamic relationships and local features between nodes.

Despite advancements, many methods struggle to fully integrate multiple feature modalities and model complex relational contexts, particularly in group interactions and higher-order semantic relationships. Addressing these challenges, this essay suggests a novel model combining GNNs and Transformer architectures for SRR. The model aims to enhance recognition accuracy and robustness in complex social relationship scenarios by effectively fusing individual, interactional, and group features.

### 2.2. Transformer-Based Relationship Modeling

Transformer-based models have significantly advanced, moving from theoretical foundations to practical applications across various fields. Vaswani et al. [26] presented the mechanism of self-attention, providing a framework for capturing long-range dependencies and overcoming limitations associated with traditional Recurrent Neural Networks (RNNs). Building upon this, Gao et al. [27] proposed an approach to connection modeling that utilizes adaptive token division, which effectively combines the advantages of dual-stream and single-stream tracking. It reduces confusion between target and background by utilizing selective interaction, attention masking, and Gumbel-Softmax for efficient training, achieving state-of-the-art performance on various benchmarks.

However, standard Transformer models face challenges in representing structured relationships, such as overlapping or nested relationships. To address this, Soares et al. [28] proposed the Virtual Token Contrastive Learning framework, incorporating learnable [REL] tokens to explicitly define relationship boundaries. Incorporating graph-structured data, Zhang et al. [29] introduced Graph-BERT, which encodes graph features, such as node degree centrality, into Transformer position embeddings, achieving an AUROC of 0.912 in protein interaction prediction tasks. Diao et al. [30] introduced a relational attention mechanism, enhancing Transformer models by integrating prior relationships between entities as attention biases. Chen et al. [31] proposed a structure-aware Transformer, augmenting the perceptual and modeling capabilities of Transformer architectures. These developments underscore the dynamic and evolving landscape of Transformer-based relationship modeling, highlighting their efficacy and adaptability across various complex relationship modeling tasks.

## 3. Methods

In this section, we introduce the GT-SRR framework. We first describe the model architecture, followed by discussions on node feature extraction, graph inference using GGNN, and relationship classification through the Transformer.

### 3.1. GT-SRR Framework Overview

**Figure 1:**
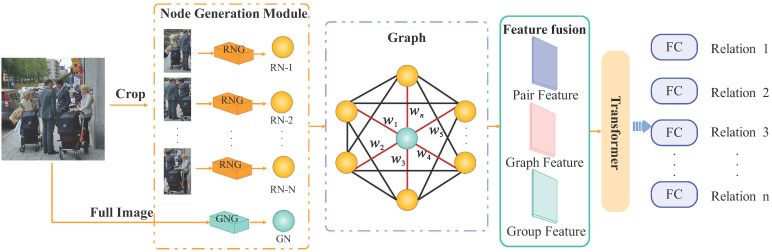
GT-SRR Framework. The GT-SRR framework comprises four stages: (1) node generation extracts relation nodes (RNs) and a group node (GN); specifically, the Relation Node Generator (RNG) encodes individual or relational features, while the Group Node Generator (GNG) captures holistic group-level representations; (2) a hierarchical graph captures local and global relational contexts; (3) multi-source features are fused for enhanced representations; (4) a Transformer-based reasoning module outputs final predictions. This unified design effectively models fine-grained interactions and global social semantics.

The GT-SRR model, similar to prior SRR models [10,13,14], focuses on pairwise relationship analysis. As shown in Figure 1, it consists of three key modules: (i) Hierarchical graph construction via hybrid feature extraction; (ii) Graph-guided relational inference; (iii) Classification via Transformer-based cross-modal fusion. Given an input image with *N* individuals, the model generates M=C(N,2) binary RNs (RN-i, i=1,…,M) and a single GN, where the group and global scene nodes are treated as one entity. Here, C(N,2) denotes the combination function, defined as $C\left(N,2\right)=\frac{N(N-1)}{2}$, which calculates the number of unique unordered pairs formed from *N* individuals.

Relation nodes are formed by combining appearance, spatial positions, and interaction features, while the group node encodes collective semantic and dynamic group characteristics. These nodes are connected via learnable weighted edges, forming a fully connected social relationship graph. A message-passing mechanism enables multi-hop reasoning between nodes: local interaction information flows from relation nodes to the group node, while global context from the group node enhances relation node representations.

The model integrates binary relationship features, graph-optimized structural features, and group features, which are processed through an enhanced MSA Transformer. This module adjusts the contribution of each feature modality, optimizing cross-level representations and effectively identifying social relationships. Higher-order feature representations are aggregated in latent space to capture key patterns. Finally, a fully connected classification layer processes the fused high-order features, yielding the social relationship probability distribution and completing recognition.

### 3.2. Node Feature Extraction

**Figure 2:**
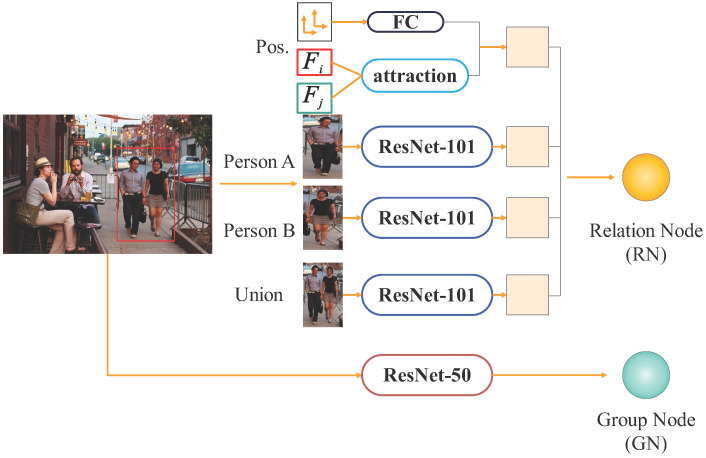
Node Generation.The node generation module extracts relation nodes (RNs) based on individual, pairwise, and positional features, and generates a group node (GN) from the global scene context to represent group-level semantics.

To capture social relationship features from images, we propose a node generation module that integrates various features to construct relationship nodes for further inference. The module architecture is shown in Figure 2.

As described in Section 3.1, for a picture that has *M* individuals, M=C(N,2) relationship nodes are generated, while only one group node is created, irrespective of the number of individuals. For example, with three individuals, three relationship nodes are formed.The node generation process for two individuals is as follows: the features of these individuals are extracted through separate channels.

As outlined in previous studies [8,13,14], the spatial distance between individuals is utilized to capture their positional and relational proximity. A completely interconnected layer is used to extract the location information, which is then encoded into the node representation. In the equation:

(1) $$\mathrm{pos}=\left\{\begin{array}{c} {}[x_{\min P_{1}},y_{\min P_{1}},x_{\max P_{1}},y_{\max P_{1}},\mathrm{area}P_{1}],\\ \left[x_{\min P_{2}},y_{\min P_{2}},x_{\max P_{2}},y_{\max P_{2}},\mathrm{area}P_{2}\right] \end{array}\right\}.$$

In this equation, $x_{\min P_{1}},y_{\min P_{1}},x_{\max P_{1}},y_{\max P_{1}}$ represent the boundary coordinates of the bounding box of $P_{1}$, and $\mathrm{area}P_{1}$ indicates the area of $P_{1}$.

Similarly, $x_{\min P_{2}},y_{\min P_{2}},x_{\max P_{2}},y_{\max P_{2}},\mathrm{area}P_{2}$ represent the corresponding coordinates and area for $P_{2}$. These features are normalized to the range of [−1, 1], using $P_{1}$’s position as an example:

(2) $$\mathrm{area}P_{1}=\left(y_{\min P_{1}}-y_{\max P_{1}}\right)\times \left(x_{\max P_{1}}-x_{\min P_{1}}\right)$$

(3) $${\mathrm{area}}_{\mathrm{norm}}=\frac{2\times \mathrm{area}P_{1}}{{\mathrm{area}}_{\max}}-1,\;{\mathrm{area}}_{\mathrm{norm}}\in \left[-1,1\right]$$

where ${\mathrm{area}}_{\max}$ represents the maximum boundary area in the dataset. Equation (1) uses this normalization for all elements. At the same time, we make reference to the attraction formula from SIF theory [32], where the interaction force between $P_{i}$ and $P_{j}$ is proportional to the attraction between them and inversely related to the square of the distance. The formula is given as:

(4) $$F_{\mathrm{att}}=\lambda F_{i}^{T}F_{j}\frac{1}{r^{2}}$$

Here, $F_{\mathrm{att}}$ denotes the attraction force, and $F_{i}$ and $F_{j}$ are the individual features of persons $P_{i}$ and $P_{j}$, respectively. λ is a constant set to 0.5. *r* represents the Euclidean distance between $P_{i}$ and $P_{j}$, which is defined as:

(5) $$r=\sqrt{{∥F_{i}-F_{j}∥}^{2}}$$

The attraction and positional information are fused via a fully connected (FC) layer and encoded as interaction features, with an output dimension of 512.

To extract individual and joint features, the ImageNet dataset is used to pre-train the ResNet-101 and ResNet-50 models. These models can capture spatial coordinates and relational semantics from visual data. The individual and joint regions are resized to 244 × 244, yielding a 4096-dimensional feature vector. This vector is further processed through the ResNet backbone to enhance relational feature representation. To improve recognition performance, feature maps from the final ResNet layer are fine-tuned for the SRR task. Additionally, group-level characteristics are extracted using a ResNet-50 model trained regarding the Places365-Standard dataset. After resizing the input image to 448 × 448 pixels, a scene feature vector with 4096 dimensions is produced. This vector is subsequently fused with individual and global image features to form a comprehensive feature representation for social relationship inference.

All ResNet models are fine-tuned to adapt to the specific characteristics of the SRR dataset. After feature extraction, additional individual features are integrated via another ResNet-101 model. Finally, a ResNet-50 network aggregates these features, producing a 512-dimensional group-level feature representation for relational reasoning.

### 3.3. Graph Inference Based on GGNN

To implement the GGNN-based graph inference, we first construct the relational graph. Since the features of individual nodes are not always equally significant, we assign different weights to the relationship and group nodes based on their relevance. In this work, the social relationship reasoning task is formulated as a graph-based inference problem, where the constructed graph G=(V,E) is an undirected graph. The node set V consists of M=C(N,2) relation nodes, each representing a pairwise relationship among *N* detected individuals, along with a single group node that encodes holistic scene-level semantics. Thus, the total number of nodes is M+1. Edges E are defined only between each relation node and the group node, resulting in a star-shaped topology where every relation node is connected to the group node via a single undirected edge. No edges exist between relation nodes. The total number of edges is therefore *M*. This graph does not incorporate explicit edge features; instead, the dependencies between nodes are captured through dynamically learned attention weights during the message-passing process.

First, we perform a layer-wise projection to obtain the initial state $h_{RN_{i}}^{\left(0\right)}$ for each relationship node and $h_{GN}^{\left(0\right)}$ for the group node, as shown below:

(6) $$h_{RN_{i}}^{\left(0\right)}=\mathrm{ReLU}\left(W_{R}X_{RN_{i}}+b_{r}\right)$$

(7) $$h_{GN}^{\left(0\right)}=\mathrm{ReLU}\left(W_{G}X_{GN}+b_{G}\right)$$

where $X_{RN_{i}}$ represents the feature vector for relationship node *i*, and $X_{GN}$ represents the feature vector for the group node. The weight matrices $W_{R},W_{G}$ and bias terms $b_{r},b_{G}$ are learned during training. The initial feature dimensions for both the relation nodes and the group node are set to 512. Specifically, each relation node feature $h_{RN_{i}}^{\left(0\right)}\in R^{512}$ is obtained by projecting the concatenated visual, positional, and interaction features of a pair of individuals into a 512-dimensional space. Similarly, the group node feature $h_{GN}^{\left(0\right)}\in R^{512}$ is derived by projecting the group feature into the same 512-dimensional space. This consistent dimensionality facilitates unified representation and efficient integration in subsequent GGNN and Transformer-based modules.

Next, we aim to aggregate information from different relationship nodes to enrich the information captured by each node, which is essential for identifying relationships. A learnable weight α is assigned, and the features from the relationship nodes and the group node are fused as follows:

(8) $$e_{i}=\mathrm{LeakyReLU}\left(\alpha^{T}\left[h_{RN_{i}}^{\left(t\right)},h_{GN}^{\left(t\right)}\right]\right)$$

where $e_{i}$ denotes the attention score, and LeakyReLU is applied to ensure that negative values do not saturate the output. The attention mechanism normalizes these values using softmax:

(9) $$a_{i}=\frac{\exp(e_{i})}{\sum_{k=1}^{M}\exp\left(e_{k}\right)},\;i=1,2,\ldots ,M$$

where $a_{i}$ represents the attention weight for each relationship node, reflecting the degree of attention each node gives to the group node. A higher value of $a_{i}$ indicates a stronger relationship with the group node.

In the graph inference phase, we incorporate message passing from the relationship nodes to the group node. This process propagates information through the graph, enhancing the semantic representation of each node based on its neighbors. After this step, the hidden states of the group node $h_{GN}$ and the relationship nodes $h_{RN_{i}}$ are updated. The update rule for the group node is:

(10) $$m_{GN}^{\left(t+1\right)}=\sum_{i=1}^{M}a_{i}W_{p}h_{RN_{i}}^{\left(t\right)}$$

where $W_{p}$ is the learned weight matrix for propagating the messages, and the feature vector is updated iteratively. After message propagation, we use the Gated Recurrent Unit (GRU) to further update the group and relationship nodes. The GRU helps integrate information from multiple hops of message passing to refine the node features continuously. The update rules are as follows:

(11) $$h_{GN}^{\left(t+1\right)}=\mathrm{GRU}\left(h_{GN}^{\left(t\right)},m_{GN}^{\left(t+1\right)}\right)$$

(12) $$h_{RN_{i}}^{\left(t+1\right)}=\mathrm{GRU}\left(h_{RN_{i}}^{\left(t\right)},m_{RN_{i}}^{\left(t+1\right)}\right)$$

where $h_{GN}^{\left(t\right)}$ and $h_{RN_{i}}^{\left(t\right)}$ represent the hidden states at the *t*-th iteration, and $m_{GN}^{\left(t+1\right)}$ and $m_{RN_{i}}^{\left(t+1\right)}$ represent the messages passed between the nodes.

This iterative process continues until the node states converge or reach a predefined number of iterations. The graph output dimension is adjusted to 512 to facilitate subsequent fusion, as it is not directly used for classification.

### 3.4. Social Relationship Modeling via Transformer

Although graph inference provides a comprehensive structural foundation, we further enhance the model by incorporating Transformer-based fusion inspired by multi-head attention (MHA) in modern natural language processing (NLP) architectures. Specifically, the Transformer component is designed to integrate diverse features for relational modeling.

**Figure 3:**
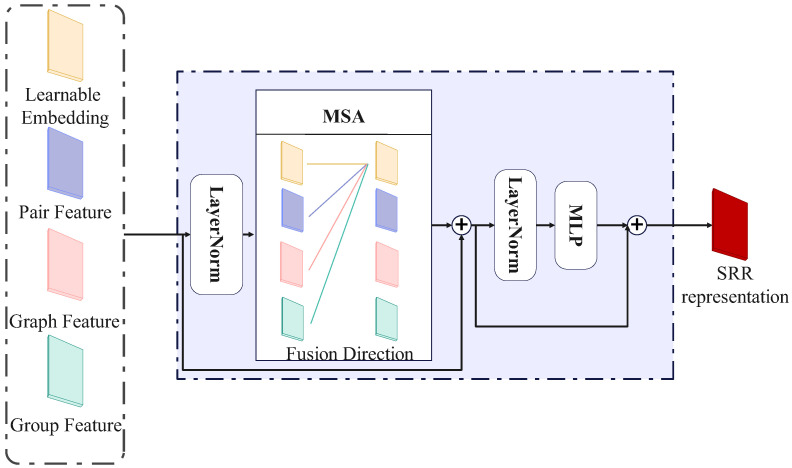
Initial Relation Modeling.This layer integrates multi-modal features and performs initial SRR via self-attention.

As shown in Figure 3, features in the model are fully utilized through interaction across visual and structural domains. The input to the feature fusion module is formulated as:

(13) $$S_{input}=\left[X_{L},X_{graph},X_{pair},X_{GN}\right]$$

where $X_{L}$, $X_{graph}$, $X_{pair}$, and $X_{GN}$ denote the learnable embeddings, graph-structural features, dyadic relational features, and group-level features respectively, each in $R^{512}$. The input sequence maintains a fixed length and dimensionality of 512, which remains unaffected by the number of individuals or the size of the graph. As a result, the Transformer module can process inputs without requiring structural adjustments for different graph sizes. This design ensures consistent cross-modal feature fusion and significantly enhances the model’s ability to capture contextual dependencies. The entire transformation process is defined as:

(14) $$S_{l}=\mathrm{MSA}\left(\mathrm{LN}\left(S_{k-1}\right)\right)+S_{k-1},\;k=1,2,\ldots ,K$$

where *K* denotes the number of Transformer layers. Multi-head attention mechanisms allow dynamic weighting of feature contributions, enabling effective fusion of global embeddings and inter-node dependencies. The final output of the Transformer encodes the social relationship representations.

In the GGNN message passing process, each node updates its features by aggregating the weighted features of its neighbouring nodes. As described in Equations (10)–(12), the weights $\alpha_{i}$ are determined by the similarity between nodes, reflecting their dependencies. However, this weighting scheme is primarily based on local neighbour information, making it difficult to capture more complex relationships between nodes and global dependencies. To address this limitation, we integrate Transformer’s MSA mechanism into the GGNN framework. To facilitate the integration, we use a projection layer to map the GGNN node features into a feature space compatible with MSA. The attention weights $\alpha_{i}$ computed by MSA are then introduced into the GGNN message passing process, dynamically adjusting the influence of each relational node on the group node. This allows the model to capture complex dependencies between nodes, overcoming the limitations of static graph structures. In particular, the dynamic attention weights $\alpha_{i}$ computed by MSA optimise the flow of information in each message-passing step. As a result, information propagation is no longer limited to local neighbour relationships, but can also capture dependencies from distant nodes, improving node feature updates. Furthermore, MSA’s multi-head mechanism enhances this process by allowing each node to focus on relationships at multiple scales, providing richer contextual information for feature updates and improving the model’s ability to capture both global and local relationships.

In this context, the GN plays a critical role. It not only aggregates information, but also influences the feature updates of the relational nodes. Specifically, during each iteration, the group node adjusts the influence of each relational node on itself through the dynamic attention weights $\alpha_{i}$ computed by MSA. This dynamic weighting allows the group node to flexibly control the contribution of each relational node to the information propagation process based on its importance. As a result, the influence of the group node on the relational nodes is refined, allowing the model to capture more complex global dependencies in the message-passing process.

We further represent the *M* pairwise relationships from an image as:

(15) $$S_{input_{inter}}=\left[r_{1},r_{2},\ldots ,r_{M}\right],\;r_{i}\in R^{512},\;i\in \left(1,2,\ldots ,M\right)$$

Analogous to the previous formulation, Equation (13) constructs the Transformer-enhanced representation of each social relationship. The MSA mechanism captures contextual similarities across all pairwise relations in an image, thereby generating informative and semantically meaningful relational embeddings—an essential component in SRR tasks. For example, an image might illustrate three types of relationships: two pairs labelled as “friends” and one pair labelled as “no relation”. In our Intra-TRM MSA module, we refine input features by aggregating similarities between the current node and other relationship representations. This process improves feature integration, enabling us to capture underlying affinities more effectively.

**Figure 4:**
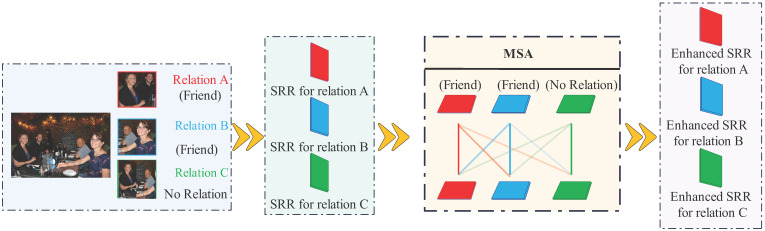
Contextual Refinement.This layer refines relational features by modeling inter-node dependencies and enhancing contextual semantics.

Notably, relationships with high internal similarity are assigned greater attention weights, while semantically dissimilar relations maintain lower affinities. This mechanism highlights important patterns while reducing confusion from visual similarities between different elements. As shown in Figure 4, this attention-guided fusion mitigates semantic ambiguity and enhances relationship discrimination.

### 3.5. Classification and Optimization

In the Transformer-based relation generation module, the final relation representation $r_{\mathrm{final}}$ is used to compute the classification probability distribution over social relation categories. The predicted probability for each class $p_{i}$ is obtained through the softmax function as:

(16) $$SR_{i}=\left\{p_{1},p_{2},\ldots ,p_{m}\right\}=\mathrm{softmax}\left(FC\left(r_{\mathrm{final}}\right)\right)$$

Here, $p_{i}$ (for i=1,2,…,m) represents the probability score for the *i*-th relation class, and *m* is the total number of relation categories. The relation types are defined according to task-specific classification schemes (e.g., PISC-C and PISC-F). SR denotes the final probability distribution of social relation classification for each instance.

We introduce a similarity-aware optimization strategy based on maximum likelihood estimation to improve classification performance further. Specifically, a Gaussian kernel–based similarity loss is employed to improve the discriminative power of relation representations. The process includes the following two aspects:

- Computing similarity between samples, including inter-class and intra-class similarity.
- Enhancing classification by aligning the predicted distribution with class-specific target distributions.

The final classification loss function is defined as:

(17) $$\mathrm{loss}=\log\left[1+\sum_{y^{\prime }\neq y}e^{z_{y^{\prime }}-z_{y}+\delta }\right]$$

where $z_{y}$ and $z_{y^{\prime }}$ represent the outputs from the final fully connected layer for the ground-truth class *y* and other classes $y^{\prime }$, respectively. The adaptive similarity margin δ is calculated as:

(18) $$\delta =\frac{num_{\max}}{num_{y}}\cdot \mathrm{cosine\_similarity}\left(y,y^{\prime }\right)$$

In this formula, $num_{\max}$ denotes the maximum number of samples across all classes in the training set, and ${\mathrm{num}}_{y}$ denotes the number of samples belonging to class *y*, while $\mathrm{cosine\_similarity}(y,y^{\prime })$ represents the cosine similarity between classes *y* and $y^{\prime }$, which reflects the semantic proximity between different relation classes and contributes to more adaptive optimization in classification.

## 4. Experiments and Results

This section introduces the datasets and implementation specifics, baseline comparisons, and ablation studies to demonstrate the efficacy of the suggested model.

### 4.1. Dataset

We evaluate our method on the People in Social Context (PISC) dataset, a widely used benchmark for SRR. The dataset contains 22,670 images and 76,568 annotated relationship samples. Following prior works, we employ a two-level hierarchical classification approach and take mAP as the primary evaluation metric. As shown in Table 1, the dataset is divided into training, validation, and test sets to ensure reproducibility and fair comparison. The coarse-level classification (PISC-C) categorizes samples into three major categories: no relation, non-intimate, and intimate. The fine-level classification (PISC-F) further refines the relationship categories into six classes: no relationship, professional, commercial, couple, friend, and family.

### 4.2. Implementation Details

Considering the class imbalance issue in the PISC-F dataset (as illustrated in Table 2), we applied data augmentation strategies to mitigate overfitting and improve model generalization. Specifically, we utilized fundamental image transformations (e.g., flipping, rotation, cropping), random occlusion, and Gaussian noise injection with a standard deviation of 0.02.

For visual feature extraction, we used pre-trained ResNet-101 and ResNet-50 as backbone networks, and incorporated a location encoder to capture bounding box coordinates and region area information. The multi-modal features—including visual, spatial, and interaction cues—were concatenated and linearly projected before passing through the graph inference module and a 4-layer Transformer encoder for relational classification.

The PyTorch 2.1 framework was used for all experiments. The model training used the Adam optimizer, starting with a 0.0001 learning rate and the OneCycleLR learning rate scheduler (peak learning rate set to 0.001). A batch size of 64 was used, and the training process spanned 100 epochs. All training and evaluation procedures were performed on an NVIDIA GeForce RTX 3060 GPU.

### 4.3. Comparative Experiments

To assess the performance of the proposed GT-SRR model, we conduct comparisons against five approaches:

Dual-Glance: The model consists of two stages that simulate human visual perception. In the first stage, it focuses on the appearance and spatial cues of the target pair to make a coarse prediction. The second stage improves these predictions by examining contextual features using attention mechanisms and region proposals, such as sual perception. The first stage attends to the appearance and spatial cues of the target pair for coarse prediction. The second stage refines predictions by exploring contextual features via attention mechanisms and region proposals.

GRM: A high-fidelity 3D reconstruction framework that recovers multi-view visual information from a single image using a pre-trained transformer backbone. It achieves accurate reconstruction within 0.1 s.

GRRN: A novel trainable GNN that constructs social relation graphs and models both pairwise relationships and contextual attributes. It employs GRUs for iterative message passing between nodes.

Graph-BERT: A Transformer-based model that incorporates BERT-style pretraining for graph-structured relational reasoning. It encodes pairwise and contextual features using attention mechanisms and achieves competitive performance without explicit graph construction.

SRT: A simple relation transformer designed for social understanding. It directly models relationship semantics through stacked Transformer layers, without relying on intermediate graph modules, but shows limitations in fine-grained categories due to lack of explicit structure modeling.

To ensure a fair comparison of performance under consistent training conditions, all baseline models were trained using the same settings as the proposed GT-SRR. Specifically, all models were implemented using the PyTorch framework and optimized with the Adam optimizer, with an initial learning rate set to 0.0001. The OneCycleLR scheduler was employed, with a peak learning rate of 0.001. A batch size of 64 was used, and training was conducted for 100 epochs. The cross-entropy loss function was adopted for all models. All experiments were carried out on an NVIDIA GeForce RTX 3060 GPU. This unified setup eliminates discrepancies due to training configurations and enables a more objective evaluation of each model’s effectiveness in social relationship recognition.

Table 3 gives the quantitative comparison of the performance of all methods on the PISC-C and PISC-F datasets. The proposed GT-SRR model achieves the highest overall mAP of 64.48% on PISC-C, demonstrating superior relational representation capabilities. Notably, GT-SRR achieves the highest accuracy of 80.10% in the Intimate category, outperforming the strongest baseline (GRRN) by approximately 1%. The advantage is even more pronounced in the Non-intimate category, where GT-SRR improves performance by over 5% compared to other models. On the more challenging PISC-F dataset, GT-SRR again achieves the best overall mAP of 64.06%, indicating its strong generalization ability across diverse relationship categories. It consistently outperforms all baselines in categories such as Friend, Family, Couple, Professional, and Commercial. For instance, in the Commercial category, GT-SRR reaches 63.06% accuracy, significantly surpassing Dual-Glance (25.71%), GRM (46.33%), and GRRN (52.63%). To further validate the effectiveness of GT-SRR, we include two representative comparison models, Graph-BERT and SRT, which adopt graph-based and rule-driven reasoning mechanisms, respectively. Graph-BERT achieves a competitive mAP of 71.30% on the PISC-C dataset, showing balanced performance across coarse-grained categories. However, its performance drops to 59.79% on PISC-F, with particularly low accuracy in the Commercial category (10.73%). In contrast, SRT demonstrates weaker overall performance, with mAP scores of 52.35% on PISC-C and 45.28% on PISC-F, and performs poorly in fine-grained categories such as Couple and Commercial. These results further underscore the robustness and superiority of GT-SRR in handling both coarse- and fine-grained social relationship recognition tasks.

These results clearly demonstrate the effectiveness of GT-SRR’s dual-stream feature fusion and hierarchical relational reasoning. Compared to Dual-Glance’s coarse ROI-based interaction modeling, GRM’s rigid geometric encoding, and GRRN’s limited GRU-based local reasoning, the improvements are evident. Graph-BERT’s BERT-enhanced graph inference with MLP fusion, and SRT’s rule-based interaction without an explicit graph structure, GT-SRR benefits from hybrid interaction modeling, hierarchical graph structure, and cross-modal attention fusion. As further detailed in Table 4, GT-SRR captures both local and global context using a hierarchical graph of relation and group nodes, and leverages GGNN-Transformer reasoning for comprehensive social relationship understanding.

To further assess performance across relation categories, we report F1-scores, which jointly consider precision and recall. The F1-score is computed as:

(19) $$F1=2\times \frac{\mathrm{Precision}\times \mathrm{Recall}}{\mathrm{Precision}+\mathrm{Recall}}$$

**Figure 5:**
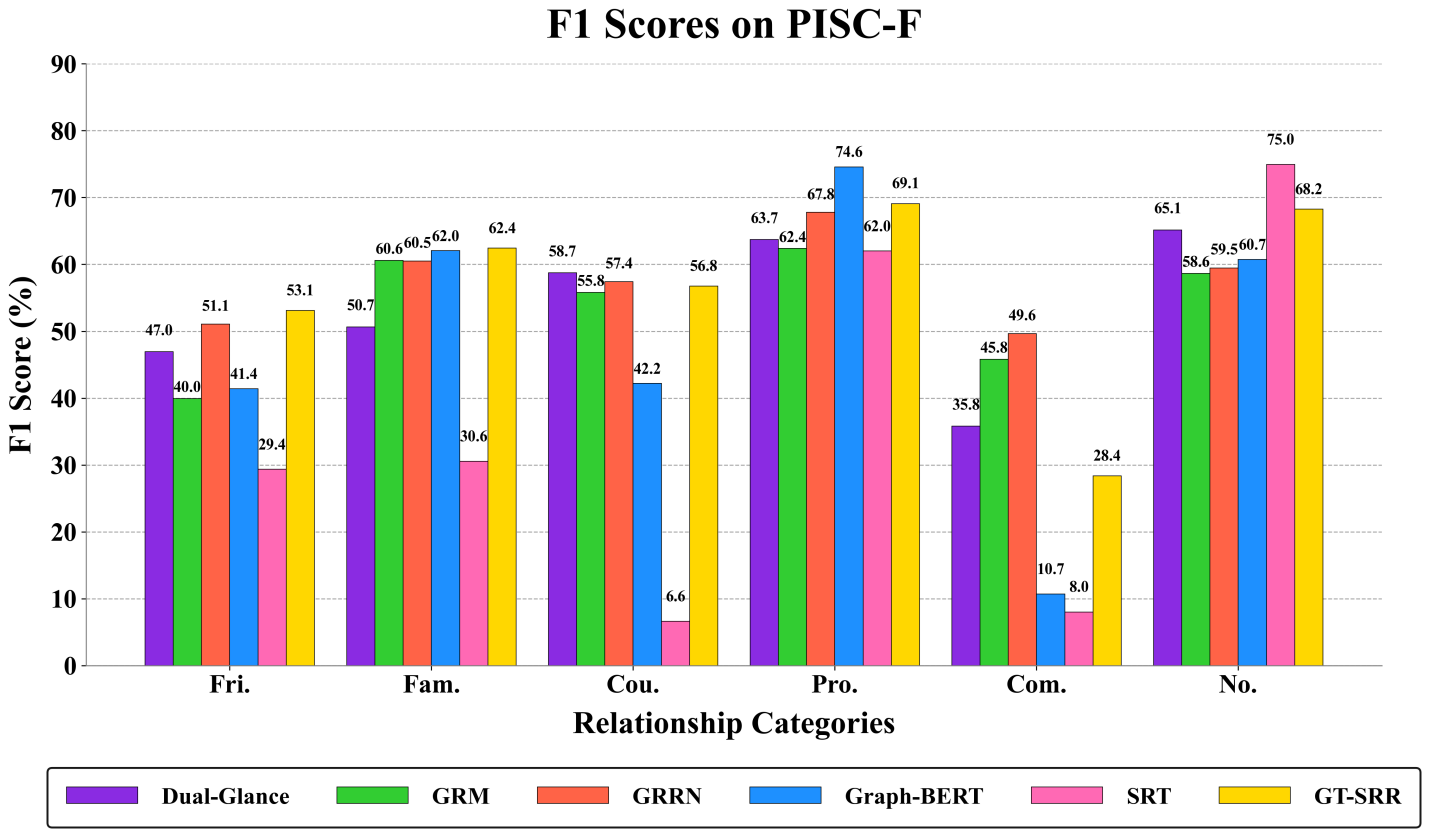
F1 score comparison of different models on the PISC-F dataset.

**Figure 6:**
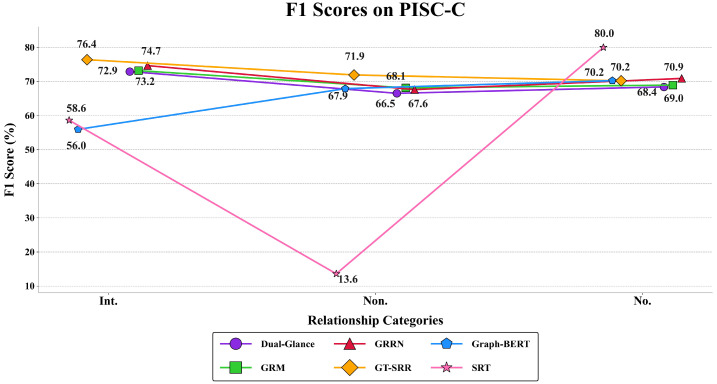
F1 score comparison of different models on the PISC-C dataset.

F1 scores provide deeper insights into model performance on different relationship types. Figure 5 and Figure 6 illustrate F1-scores across PISC-C and PISC-F, showing GT-SRR’s superiority in the categories of Intimate, Non-intimate, Friend, Family, Professional, and Commercial.

**Figure 7:**
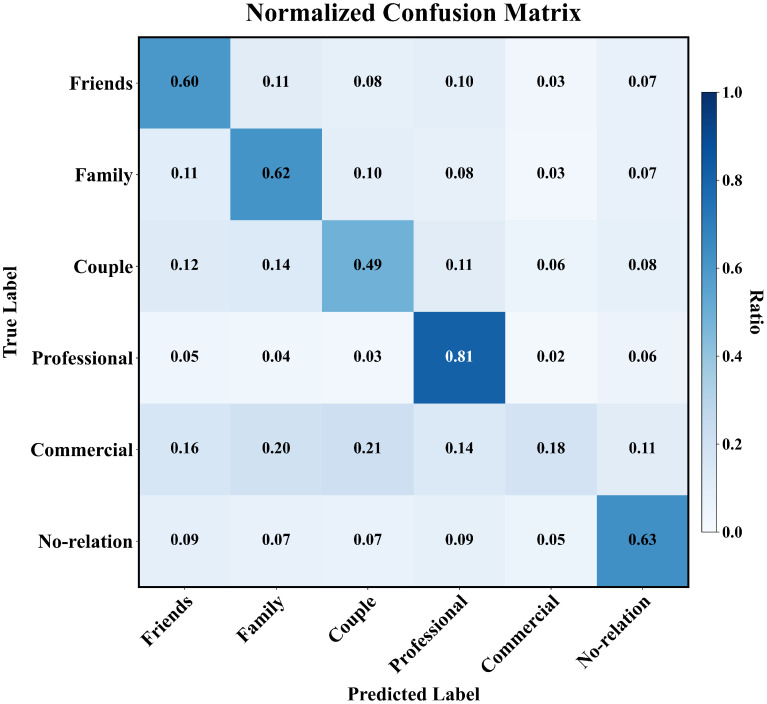
GT-SRR Confusion Matrix.GT-SRR shows high accuracy in most classes, with occasional confusion among similar relation types such as Friends, Family, and Couple.

To explore class-level prediction errors, we further visualize the confusion matrix in Figure 7. GT-SRR achieves the highest recognition rate of 81% for the Professional class, but it suffers from some misclassification between Couple–Commercial and Friends–Family due to overlapping semantics. Commercial relations are often confused with Friends due to shared visual-textual contexts. There is a classification error between the Couple and Commercial categories. The underlying cause of this error may lie in the visual similarity between the two categories, as well as a potential data distribution bias, which makes it challenging for the model to accurately differentiate between the two. These observations indicate that nuanced context modeling is essential for clarifying ambiguity in SRR. Future research should integrate more advanced semantic embeddings to enhance disambiguation abilities.

**Figure 8:**
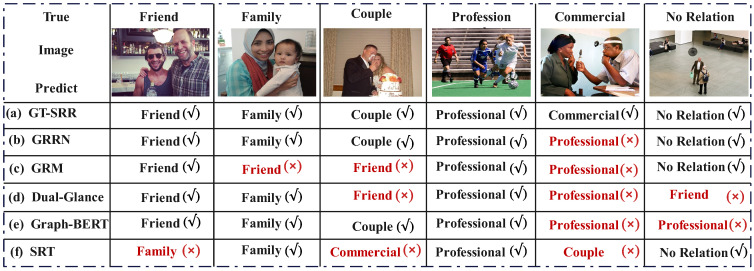
Examples of confused identification by different models. This figure presents visual examples comparing predicted relation categories across different models. Each column shows one ground-truth relation type, along with corresponding model predictions and correctness.

To better understand the differences in prediction results across models and the underlying causes of misclassification, we present representative visual samples of each relationship category in the PISC-F dataset and their corresponding recognition results, as illustrated in Figure 8. Visual analysis shows that the proposed GT-SRR model successfully identifies complex categories such as Family and Couple, whereas the GRM and Dual-Glance models demonstrate significant misclassification issues, including incorrect predictions of Familyrelationships as Friend, and Couple relationships as Friend. Additionally, the GRRN model tends to misclassify Commercial relationships as Professional. We further observe that Graph-BERT, although capturing Friend and Family relations correctly, misclassifies No Relation as Professional, indicating challenges in distinguishing contextual independence. Similarly, SRT incorrectly identifies Couple as Commercial and Friend as Family, showing limitations in fine-grained relation understanding. This analysis highlights the GT-SRR model’s superior capability in capturing subtle category boundaries, demonstrating its enhanced sensitivity and accuracy in SRR tasks, and further demonstrating the effectiveness of the proposed approach.

### 4.4. Ablation Study

This seeks to confirm the efficacy of the suggested multi-modal fusion strategy and graph reasoning mechanism. The findings are displayed in Table 5. The results show that using only the GGNN module yields an mAP of 55.54%, but it performs poorly in the non-intimate category (30.11%). The standalone Transformer module performs slightly better, with an mAP of 54.07%. When combining GGNN and Transformer without incorporating group features, the performance improves substantially to an mAP of 74.84%, indicating the benefit of structural and attention-based fusion. However, the whole model—integrating GGNN, Transformer, and group-level features—achieves the best overall performance, reaching an mAP of 80.52%, with a significant gain in the intimate category (73.69%). These results confirm the complementary strengths of the two modules and highlight the critical role of group-level semantics in enabling more effective modeling of spatial and contextual relationships.

To further validate the role of GGNN in the GT-SRR framework, we replaced the GGNN with a Graph Convolutional Network (GCN) and a Graph Attention Network (GAT), while keeping all other settings consistent. The results of this comparison are presented in the Table 6.

**Figure 9:**
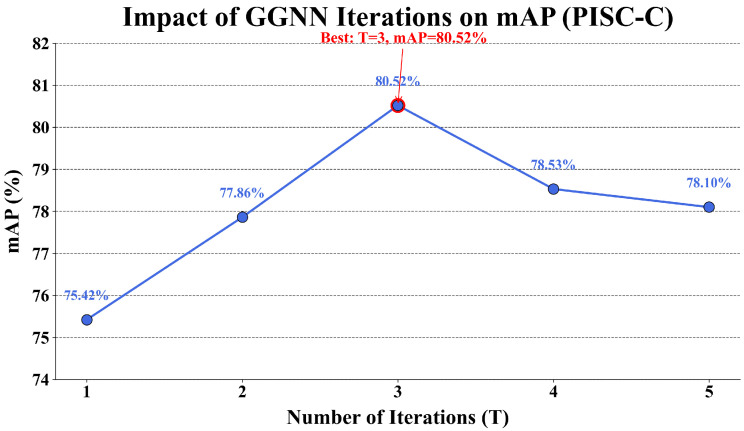
Impact of GGNN iterations on mAP in the PISC-C dataset.

We evaluate the model under different iteration settings to investigate the impact of the number of GGNN iterations. As shown in Table 7 and Figure 9, the best performance is achieved when T = 3, with an mAP of 80.52%. Fewer iterations result in insufficient feature aggregation, while excessive iterations may lead to overfitting.

To further investigate the impact of different feature fusion strategies, we conducted ablation experiments on the PISC-C dataset, comparing four configurations: simple concatenation, attention-based fusion, gated fusion, and our proposed multi-head self-attention (MSA) fusion. The results are presented in Table 8. As shown, the gated fusion method achieves relatively high accuracy in the Intimate category (80.62%), but its performance in the Non-intimate and No-relation categories is less competitive. Attention-based fusion performs more consistently across all classes and outperforms simple concatenation. Most notably, our proposed MSA-based fusion demonstrates superior performance across all relationship categories. In particular, it significantly boosts accuracy in the Non-intimate category to 73.69%, while achieving the highest overall mAP of 80.52%. This confirms that the MSA module effectively captures global context and fine-grained dependencies, offering a more robust and expressive representation for social relationship recognition than traditional fusion methods.

## 5. Conclusions

In this research, we propose GT-SRR, a context-aware framework for social relation recognition that integrates GGNN and Transformer modules. The model effectively captures individual attributes, interaction cues, and group-level context through a hybrid feature extraction mechanism. By constructing relation and group nodes and modeling their structural dependencies with GGNN, the framework enables local interaction reasoning. Additionally, a Transformer encoder performs global context modeling and captures higher-order semantic associations. Incorporating group semantics provides complementary contextual information that enhances the robustness of pairwise relation inference. Experimental results on the PISC dataset demonstrate that GT-SRR achieves superior performance compared to existing methods, particularly in complex relational scenarios. This work advances multi-feature fusion and hierarchical relation modeling in social relation recognition tasks.

## Figures and Tables

**Table 1:** Task split statistics for different SRR levels.

	Training	Validation	Testing
PISC-C	13,142/49,017	4000/14,536	4000/15,497
PISC-F	23,207/72,612	500/1505	1250/3961

Note: 16,826/55,400 indicates that 16,826 training images correspond to 55,400 social relation samples in PISC-F.

**Table 2:** PISC-F Fine-level Sample Distribution.

Rel.	Fri	Fam	Cou	Pro	Com	No
Samples	12,686	7818	1552	20,842	6523	11,979

Note: Fri: Friend; Fam: Family; Cou: Couple; Pro: Professional; Com: Commercial; No: No relation.

**Table 3:** Performance Comparison on PISC-C and PISC-F Datasets.

Model	PISC-C				PISC-F						
	Int.	Non.	No.	mAP	Fri.	Fam.	Cou.	Pro.	Com.	No.	mAP
Dual-Glance	78.25	65.04	63.99	76.40	56.51	39.44	63.92	75.06	25.71	59.75	57.15
GRM	76.12	68.28	68.13	74.84	38.14	60.41	61.18	67.72	46.33	54.97	56.45
GRRN	79.08	64.25	69.36	77.30	58.67	57.61	77.25	64.10	48.59	52.63	63.17
Graph-BERT	55.95	67.90	70.24	71.30	41.39	62.04	42.19	74.59	10.73	60.72	59.79
SRT	58.62	13.58	79.97	52.35	29.37	30.58	6.64	62.00	8.03	74.95	45.28
GT-SRR (Ours)	80.10	73.69	64.48	**80.52**	60.08	61.60	48.63	81.12	18.08	63.06	**64.06**

Note: We report per-class accuracy (%) and mean Average Precision (mAP, %) for PISC-C and PISC-F datasets. Int. = Intimate, Non. = Non-intimate, No. = No-relation. Best results are highlighted in bold.

**Table 4:** Structural Comparison of GT-SRR and Baseline Models.

Component	Dual-Glance	GRM	GRRN	Graph-BERT	SRT	GT-SRR (Ours)
Individual Feature Extraction	✓	✓	✓	✓	✓	✓
Interaction Modeling (Position + Behavior)	ROI-based Glance Module	3D Geometric Encoding	GRU-based Edge Modeling	Graph-Based Interaction	Simple Distance Rule	Hybrid Interaction
Scene Context Integration	–	✓	✓	✓	✓	✓
Group Semantics Modeling	–	–	–	–	–	✓
Feature Fusion Strategy	Concat- enation	Geometric-Spatial Fusion	Contextual Feature Fusion	MLP-based Fusion	Direct Concatenation	Cross-modal Attention Fusion
Graph Structure (RNs + GN)	–	–	Static Graph	BERT-Enhanced Graph	–	Hierarchical Graph
Relational Reasoning Method	–	–	GRU-based Local Reasoning	Graph-BERT Layers	Rule-based Prediction	GGNN + Transformer

Note: ✓ indicates the model includes the corresponding component. The proposed GT-SRR introduces hierarchical graph reasoning and integrates both local and global contextual modeling, achieving a more comprehensive social relationship representation compared to existing models.

**Table 5:** Performance comparison of individual modules and their combination on PISC-C.

	Int.	Non.	No.	mAP
GGNN	70.02	30.11	61.52	55.54
Transformer	68.93	24.18	64.16	54.07
GGNN+Transformer (without group feature)	83.09	52.41	62.03	74.84
GGNN+Transformer+group feature (ours)	80.10	73.69	64.48	80.52

**Table 6:** Comparison of different graph reasoning modules on PISC-C.

	Int.	Non.	No.	mAP
GCN	66.62	67.90	70.08	73.52
GAT	64.75	70.26	68.70	74.17
GGNN	80.10	73.69	64.48	80.52

**Table 7:** Comparison of mAP performance with varying GGNN iterations on PISC-C.

Iterations (T)	1	2	3	4	5
mAP (%)	75.42	77.86	80.52	78.53	78.10

**Table 8:** Comparison of Different Feature Fusion Strategies on PISC-C.

Fusion Method	Int.	Non.	No.	mAP
Simple Concatenation	72.12	45.33	61.49	70.35
Attention-based Fusion	78.37	52.84	68.00	73.24
Gated Fusion	80.62	56.20	56.84	73.97
MSA (ours)	80.10	73.69	64.48	80.52

## Data Availability

The People in Social Context (PISC) dataset is available at https://zenodo.org/records/1059155 (accessed on 19 January 2025).
